# Retraction Note: A novel polymorphism of the GP78 gene is associated with coronary artery disease in Han population in China

**DOI:** 10.1186/s12944-015-0063-9

**Published:** 2015-07-23

**Authors:** Erdenbat Cha, Zhen-Yan Fu, Yi-Tong Ma, Qing Zhu, Xiang Xie, Fen Liu

**Affiliations:** 1 Department of Cardiology, First Affiliated Hospital of Xinjiang Medical University, Urumqi, Xinjiang 830054 China; 2 Xinjiang Key Laboratory of Cardiovascular Disease Research, Urumqi, Xinjiang 830054 China

This article [1] has been retracted by the authors due to extensive overlap with previously published work, most notably Zhu *et al.*, Liu *et al.*, and Fu *et al.* [2–4]. The authors apologise for any inconvenience caused.
